# Translational data analytics in exposure science and environmental health: a citizen science approach with high school students

**DOI:** 10.1186/s12940-020-00627-5

**Published:** 2020-07-01

**Authors:** Ayaz Hyder, Andrew A. May

**Affiliations:** 1 Division of Environmental Health Sciences, College of Public Health, The Ohio State University, 1841 Neil Ave., Cunz Hall, Room 380D, Columbus, OH 43210 USA; 2 Translational Data Analytics Institute, The Ohio State University, 1841 Neil Ave., Cunz Hall, Room 380D, Columbus, OH 43210 USA; 3 Department of Civil, Environmental and Geodetic Engineering, College of Engineering, The Ohio State University, 2070 Neil Avenue, 483A Hitchcock Hall, Columbus, OH 43210 USA; 4 Ohio State University Center for Automotive Research, 2070 Neil Avenue, 483A Hitchcock Hall, Columbus, OH 43210 USA

**Keywords:** Air pollution, Low-cost sensors, Citizen science, Translational data analytics

## Abstract

**Background:**

Translational data analytics aims to apply data analytics principles and techniques to bring about broader societal or human impact. Translational data analytics for environmental health is an emerging discipline and the objective of this study is to describe a real-world example of this emerging discipline.

**Methods:**

We implemented a citizen-science project at a local high school. Multiple cohorts of citizen scientists, who were students, fabricated and deployed low-cost air quality sensors. A cloud-computing solution provided real-time air quality data for risk screening purposes, data analytics and curricular activities.

**Results:**

The citizen-science project engaged with 14 high school students over a four-year period that is continuing to this day. The project led to the development of a website that displayed sensor-based measurements in local neighborhoods and a GitHub-like repository for open source code and instructions. Preliminary results showed a reasonable comparison between sensor-based and EPA land-based federal reference monitor data for CO and NOx.

**Conclusions:**

Initial sensor-based data collection efforts showed reasonable agreement with land-based federal reference monitors but more work needs to be done to validate these results. Lessons learned were: 1) the need for sustained funding because citizen science-based project timelines are a function of community needs/capacity and building interdisciplinary rapport in academic settings and 2) the need for a dedicated staff to manage academic-community relationships.

## Introduction

Translation of environmental health science from data to knowledge to action is valuable on several different levels and is a major theme of the 2018–2023 Strategic Plan for NIEHS [1]. At the individual level, translation may be characterized as the monetary and non-monetary benefits individuals may receive, for example, clean air to breathe and safe water to drink. At the community level, translation may refer to outreach and educational opportunities for students, vulnerable populations, and the general public. At the societal level, translation may manifest as the overall impact of environmental health science research in terms of reducing adverse outcomes associated with environmental and occupational exposures [2]. Additional factors that enable the process (i.e. turning data to knowledge to action) and create value (i.e., benefits to individuals, community, and society) of translation at each of these levels include i) ability and capacity for interdisciplinary research/training and ii) community engagement [3, 4]. In light of Frumkin’s call for consequential environmental epidemiology [5], which emphasizes the translation of knowledge to action, translational research in environmental health sciences must pay close attention to both of these enabling factors—community engagement and interdisciplinary research.

Community engagement is defined in many ways given the definition of “community” and the type of engagement that takes place. On the one hand, there is community-engaged research (CEnR) where a defined group of individuals in a geographic location are engaged from the start to the end of the research process [6]. Examples of CEnR include community-based environmental monitoring studies, such as Imperial County Community Air Monitoring Network [7] and environmental justice studies [8]. Citizen science is another form of participatory research where citizens are recruited to assist with data collection efforts in a scalable manner that would not have been cost-effective or logistically possible [9]. Community engagement may also take the form of academic-community partnerships where academic institutions are invited by or approach afflicted communities to research the issues and/or develop and implement interventions. A good example is the Deep Water Horizon disaster where several academic-community partnerships were formed to address environmental health issues in afflicted communities and populations [10]. We focus on community engagement that fits between citizen science and academic-community engagement where interactions take place between researchers and staff from a University or other academic institution and the community. These types of engagements typically take place outside the University setting (i.e., physical premises). Within the University setting, interdisciplinary research takes place involving two or more researchers from different disciplines. Interdisciplinary research is defined in a National Academies’ report as “a mode of research by teams or individuals that integrates information, data, techniques, tools, perspectives, concepts, and/or theories from two or more disciplines or bodies of specialized knowledge to advance fundamental understanding or to solve problems whose solutions are beyond the scope of a single discipline or area of research practice” [11]. The interactions between researchers from across disciplines may be facilitated by opportunities involving funding, intellectual curiosity or a request from a community partner that requires an interdisciplinary team to address the complex issue.

**Fig. 1:**
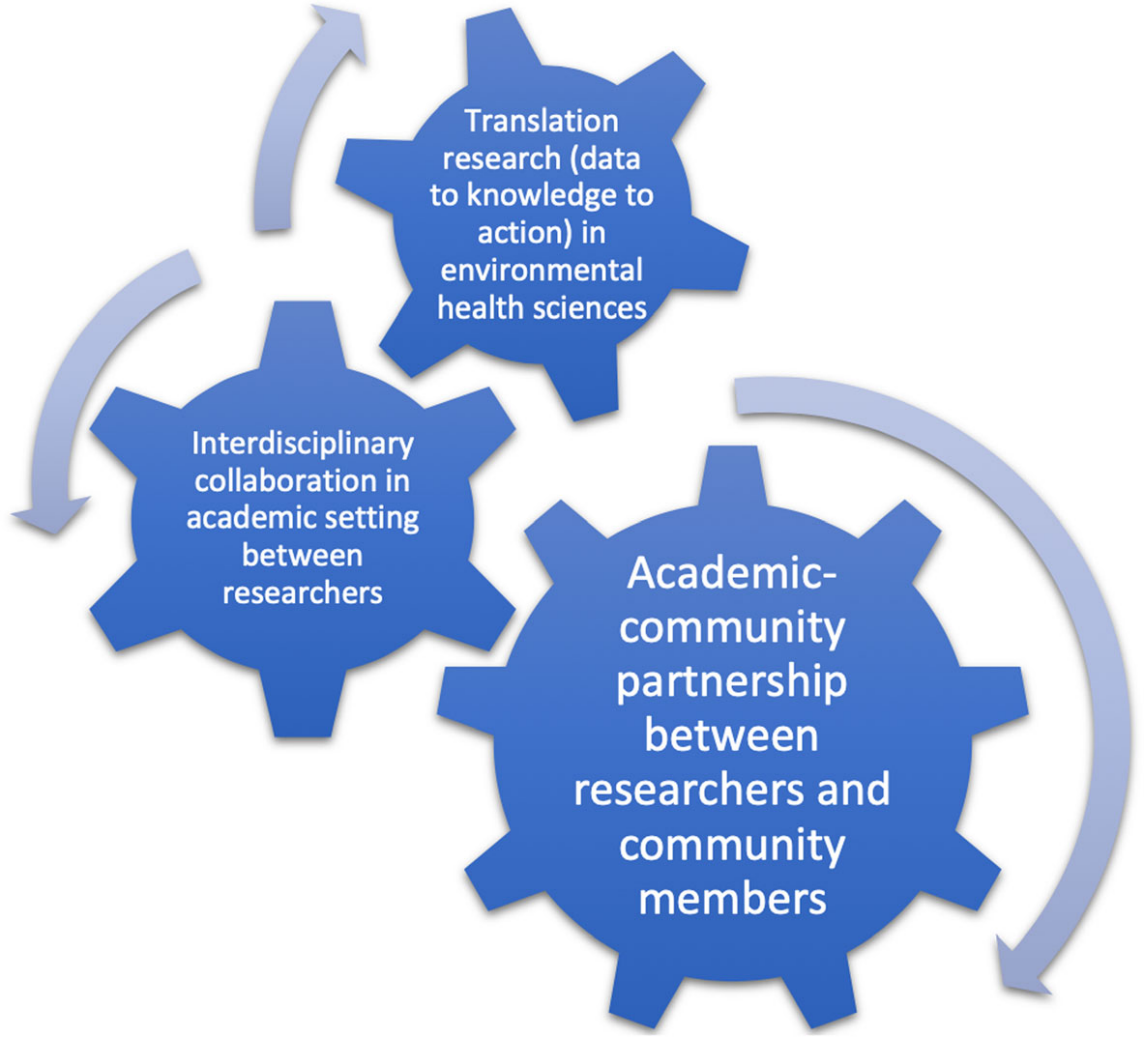
Schematic showing how community engagement and interdisciplinary research drive each other and in-turn affect translational environmental health sciences research

Based on these definitions of community engagement and interdisciplinary research, we can identify interactions between the setting (e.g. University or community) and actors (e.g., academic researchers, school teachers, high school students, public health officials) involved within each of these two enabling factors via a schematic (Fig. 1). This schematic illustrates how each factor (community engagement and interdisciplinary research) continuously interacts with each other. Ultimately, these interactions “turn the wheels” of translational research in environmental health. A challenging aspect of translational research, whether in clinical medicine or environmental health sciences, is the integration of multiple processes along the continuum of translation from data to knowledge to action. Translational data analytics (TDA) for environmental health is a good candidate for such an integration.

Translational data analytics is a new term for an emerging field that applies data analytics principles and techniques to disciplines to bring about broader societal or human impact. TDA for environmental health is an emerging discipline that has the potential to operationalize the processes of translation and integrate translation with community engagement in a unique manner. The emergence of TDA, in general, stems partly from a desire for “Big Data” methods and technologies to be of consequence and justify the public and private investments in Data Science and “Big” data analytics [12, 13]. In the same vein, current citizen-science based approaches also make use of recent technological shifts in the form of smartphones, apps and low-cost sensors to enable large-scale and ongoing data collection [6, 9]. This is a shift from traditional citizen-science approaches, which emerged from conversation biology (e.g., annual Audubon Christmas Bird Count). Emerging trends in citizen science for environmental health research indicate that technology will continue to play a major role in addressing the changing needs of citizens and communities [14] although it is not clear how citizen science-based approaches can contribute to translational research and policy changes [6].

In environmental health sciences, the use of data-intensive methods and technologies is slowly emerging and recent examples include computational toxicology, computational exposure science, linked data platforms to inform environmental health [15, 16], and Artificial Intelligence-based technologies [17, 18]. While these advances are reasonably translating data to knowledge to action and spurring interdisciplinary collaborations within the academic setting, there is a lack of emphasis or attention paid to community engagement, especially academic-community collaborations. Paying little or no attention to community engagement in any effort of translational data analytics for environmental health risks diminishing the value, benefits and sustainability of such efforts over time. Therefore, the gap in knowledge to be addressed is how to integrate interdisciplinary collaborations and academic-community engagements within the emerging discipline of TDA for environmental health. The primary objective of this study is to describe a real-world example of an environmental health translational data analytics project.

## Methods

### Project overview

On its most fundamental level, this project is a facilitated partnership between an academic institution (The Ohio State University, OSU) and a high school engineering class (Engineering Design and Development (EDD) Course offered at Hilliard Davidson High School). The structure of the partnership is more complex because of its interdisciplinary nature. At the academic institution, which will be referred to as OSU, faculty members were from the College of Public Health (author A.H.) and College of Engineering (author A.A.M.). Hilliard, OH is a suburb in the Greater Columbus Area with a median household income of $95,831 (2018 dollars) and predominantly White (88%), educated (95% high school graduate or higher) and employed (70%) population [19]. higherA brief overview of the project is described below followed by a description of the project structure, role and responsibilities of each partner and details on the process of carrying out the project.

In this citizen science project, Wi-Fi-enabled, low-cost air quality sensors were fabricated and deployed by a team of citizen scientists comprised of local high school students. Sensors focused on traffic-related air pollution (TRAP), namely carbon monoxide, ozone (O_3_), nitrogen dioxide (NO_2_), and particulate matter (PM). A cloud-computing solution was developed and integrated with the sensor network deployment process to provide publicly available, near-real-time air quality data for risk screening purposes and curricular activities. Jointly, the citizen scientists and academic team developed training modules for teachers and other students to replicate the sensor deployment process and developed curriculum activities targeting middle school grades based on data collected from the sensors. The deployed sensors monitored ambient air quality at school buildings maintained by the Hilliard City School District. Also, outreach activities about local air quality were led by the high school students within their neighborhoods where they live, play and go to school.

The deployed network of air quality monitors provided estimates of exposure to air toxicants within key micro-environments in the suburb of Hilliard, OH. Potential public health applications of the sensor network, which are envisioned for future projects, include environmental education and supporting prevention and management strategies for acute asthma exacerbation among susceptible populations (e.g., smartphone apps with real-time alerts based on local air quality measurements from sensor network). By providing a cloud-computing solution to process the data collected by the sensor network, biases and outliers in exposure estimates were assessed, thus mitigating some of the concerns related to data reliability, validity, and trustworthiness.

At the beginning of this facilitated partnership, we discussed several concerns with our high school partners (teachers and students) regarding air quality measurements. First, we clarified that the use of low-cost sensors did not meet the rigorous scientific standards of air pollution exposure assessment for health effects studies and that what was being measured by the sensors was not directly relatable to EPA land-based Federal Reference Monitors [20]. This explanation was not well understood by high school partners and required repeated explanations over the course of the multi-year project. Also, this limitation of sensors did not affect the goal of this environmental health translational data analytics project because we had no plans to conduct health effects studies in the short-term and were primarily focused on working out the processes for deploying a sensor network.

**Fig. 2:**
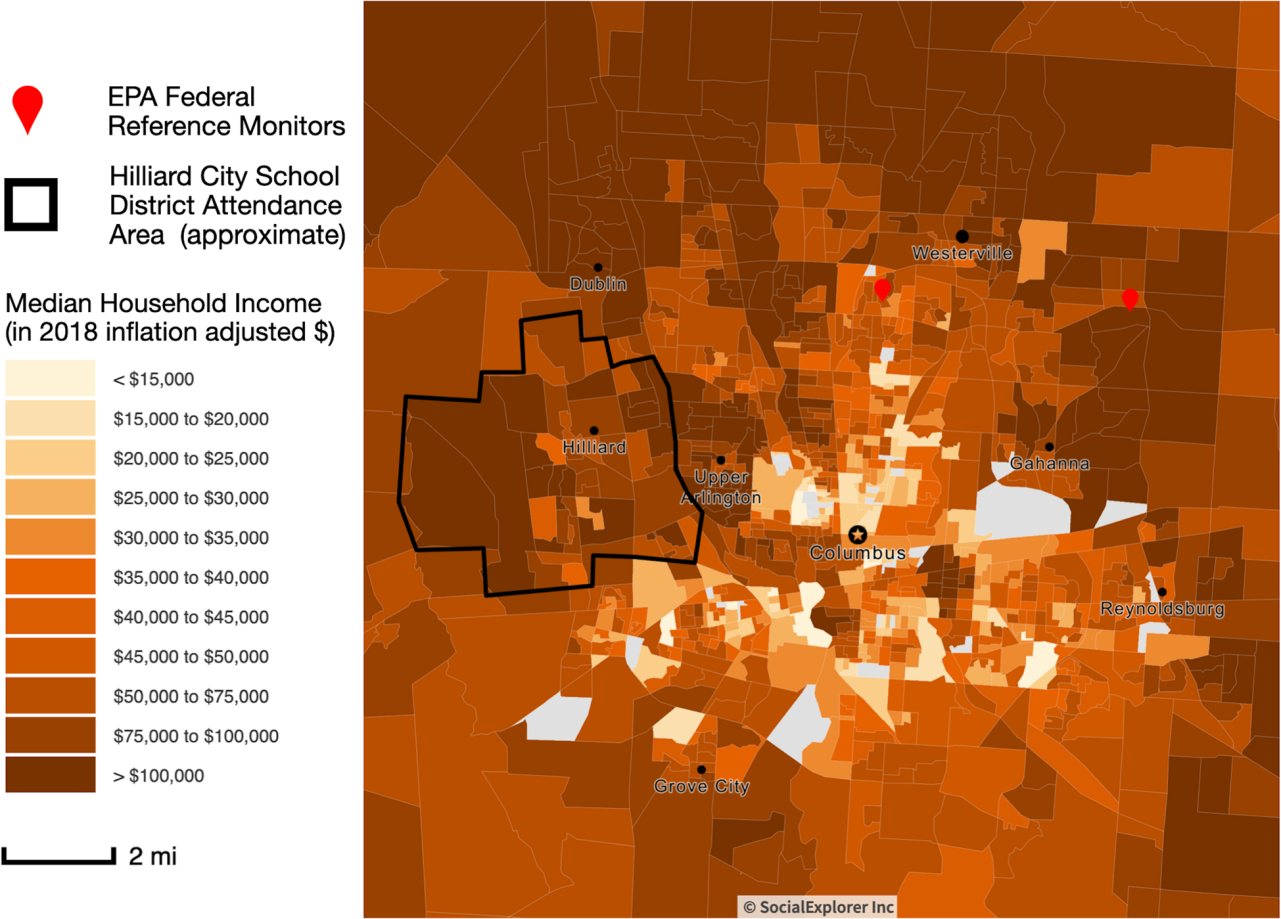
Map of school buildings in the Hilliard City School District. Potential sites for deployment of low-cost air quality sensors are circled on the map. This map was developed in Year 1 of the project and underwent revisions over the course of the project

Second, we explained the reliability of air quality sensors by discussing the need for calibration, maintenance and environmental conditions under which sensors operate. These explanations were conveyed over the course of the partnership as each cohort of students tackled different aspects of the project. For example, when students were choosing different materials for the sensor housing unit, we explained the impact of wind, rain and sunlight on each sensor. We did not perform co-location studies of the low-cost sensors and instead calibrated the sensors in one of the co-authors (A.A.M.) environmental engineering lab in order to have some sense of reliability. Uncertainty in sensor measurements was explained as being due to equipment quality and weather conditions. On the web-based application for visualizing sensor-based data a moving average was plotted to account for uncertainty and outliers in the calibrated data coming off the sensor. The window of time over which to smooth the data was user-selected and allowed students the opportunity to interactively manipulate the data. Also, we plotted the NAAQS standard for each air toxicant as a way of checking that sensor-based data were at least in a reasonable range of values when compared to the NAAQS standard values. Since the EPA monitors were located outside of the study area (Fig. 2, red drops) we also explained to students how a direct comparison between sensor-based measurements in Hilliard and EPA monitors nearby was not appropriate for some air toxicants more than others. For example, regional levels of air toxicants, such as PM, were more relevant for such as comparison than more localized levels of NO_2_.

Lastly, we discussed a concern brought up by the high school teachers and students about whether Ohio EPA needs to be informed about any sensor-based measurements that exceed NAAQS standards. The academic faculty consulted with Ohio EPA Air Quality Division staff and confirmed that sensor-based measurements did not meet the criteria for reporting exceedances to their agency. Along similar lines another question arose about whether the school and school district administrators would need to take any corrective or mitigation actions if sensor-based measurements exceeded NAAQS standards. The academic faculty worked with concerned school administrators, teachers and students to reassure them that sensor-based measurements should be viewed as risk screening tools and the limitations of the data did not rise to the level of having to take any additional actions.

### Role and responsibilities of partners

#### Academic faculty

Among the academic faculty, AH had experience with air pollution exposure assessment, knowledge of air pollution-related health outcomes and disparities and technical skills for cloud computing and data analytics and AAM had to experience with the engineering design process, knowledge of air quality sensors and technical skills for sensor fabrication and calibration. These experiences, knowledge and technical skills determined the role and responsibilities of each faculty member in the project. AAM mainly provided mentoring and guidance to students with sensor fabrication, including but not limited to Wi-Fi connectivity, solar-based power requirements, and engineering design principles. AH mainly guided where to deploy sensors based on maps of population density, land use and socioeconomic data and computer programming and web-based application development and testing. The faculty served as both “clients” and “consultants” for the students’ year-long engineering design projects in that the students worked to meet the “client” demands, but the students also have access to the available resources from the “consultants”, such as equipment for sensor testing and calibration, to conduct the work.

#### High school teachers

Initially, the academic faculty were connected with two high school engineering teachers at the high school. After initial conversations about the nature of the project, one teacher, who taught the EDD course, agreed to participate. The key considerations for the suitability of the teacher experienced with external partnerships (i.e., external to the high school) and the engineering/public health focus of the project. In recent years, a student who attended Davidson High School had died of an asthma attack and so the teacher was also attracted to the project’s focus on traffic-related air pollution. The teacher had previous experience running an apprenticeship program with a local technical training program (Tolles Career & Technical Center). The learning objectives of the EDD course lent itself naturally to the goals of the proposed academic-community partnership in terms of being student-led, engineering-focused on societal relevance and emphasis on interdisciplinarity. The primary role of the teacher was to facilitate interactions with students and school/district administrators and ensure learning objectives were met by students for the EDD course. The teacher also facilitated the ongoing recruitment of students for subsequent cohorts over the multi-year project. Initially, we (the academic faculty) planned to co-develop curricular materials with high school teachers based on the collected air quality data but this responsibility was transferred solely to the high school teachers given their greater familiarity with curricular standards and expectations. Teachers were reimbursed for their effort in developing curricular materials.

#### High school students

To date, four cohorts of students from the EDD class participated in the project with a new cohort each school year. Students for the first cohort were identified by the EDD teacher and students in subsequent cohorts were recruited by the previous years’ student cohort. Within each cohort, the student’s self-selected roles and responsibilities. For example, one student each year was primarily a “project manager” who was responsible for documentation of project tasks and communication with OSU faculty. Some students pursued more “physical” roles including fabrication and testing, while others were more “computational” including 3D modeling and programming.

### Details on the process of carrying out the project

The following set of processes were used to carry out this translational data analytics project in environmental health. The order of processes varied based on the timeline of the project. OSU faculty met with students regularly (at least once per month, but sometimes more frequently) during the academic year to discuss progress and provide guidance on matters outlined in the processes below. Some of these were “work sessions” including sensor package fabrication and code debugging, while others were more related to planning.

**Fig. 3:**
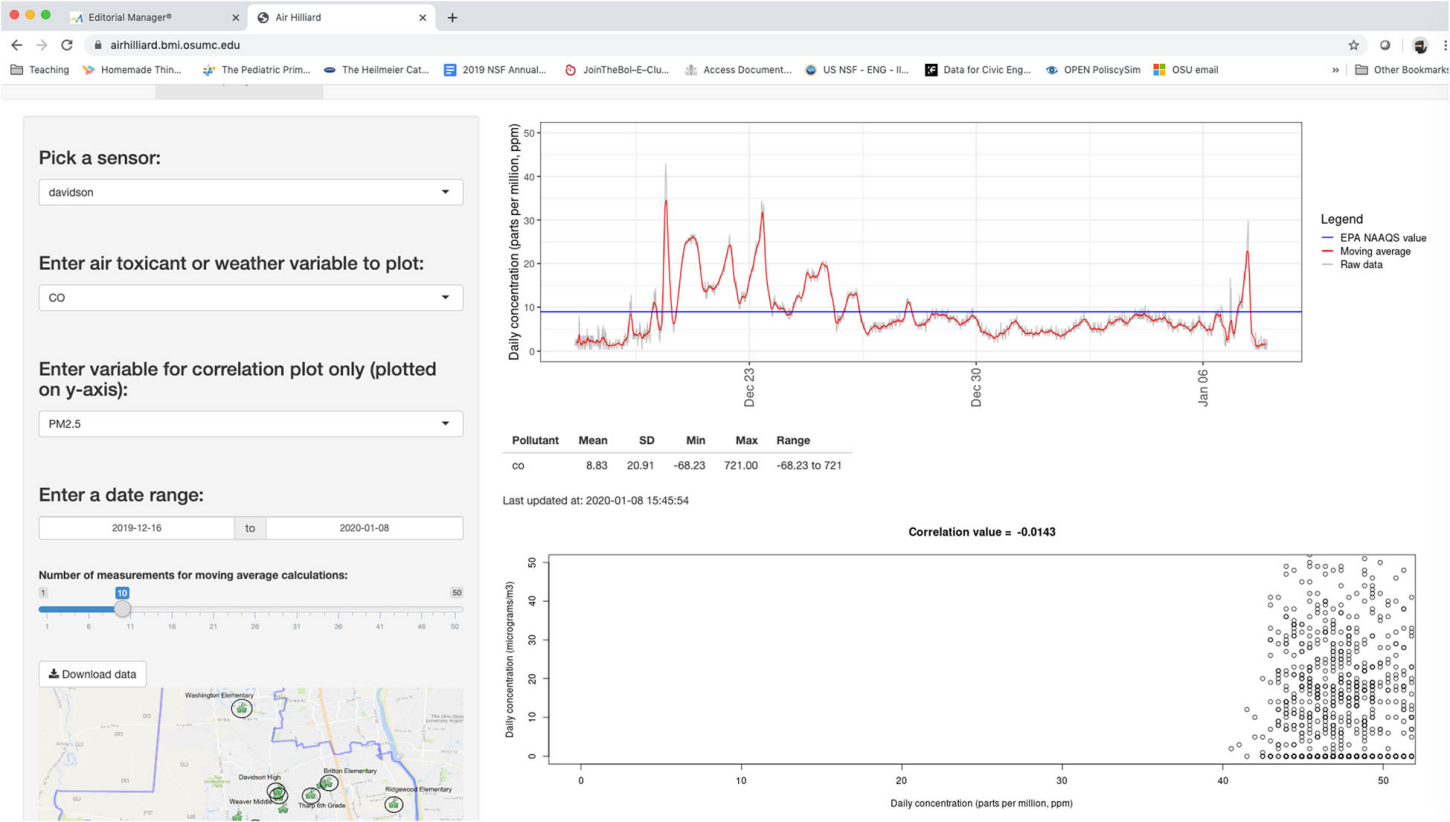
Screenshot of the website developed for visualizing sensor-based data

1. Introduction: Partners met in-person to learn about the overall project, discuss roles and responsibilities, align vision and objectives between the educator (high school teacher for EDD), students (cohort in EDD high school class) and academic researchers (for academic faculty) and identify tasks with timelines.
2. Education: Both partners learned about each other’s capabilities and resources, in terms of materials and equipment for sensor fabrication, the design and production of sensor enclosure, and computer programming, and shared knowledge, in terms of rules and regulations regarding access to local area schools (e.g., rooftop, Wi-Fi, and after-hours access) and which schools to prioritize for deploying a complete sensor package (Fig. 2). This prioritization process involved academic researchers educating students about the basics of atmospheric sciences as it related to exposure assessment, the influence of road types (e.g., highways, major arteries, local roads), wind direction and the association between socioeconomic disparities and health outcomes by reference to epidemiological studies. High school students were shown maps of demographic, social and economic variables (Fig. 3 is an example of median household income) to demonstrate the heterogeneous characteristics of the population. Satellite-based images from Google Maps were used to visually assess each school building’s proximity to major roadways. Additional factors, such as proximity of industrial areas and traffic roundabouts, were also explained as important factors to consider for measuring local scale air quality in the study area. High school students educated academic researchers about the logistical issues involved with accessing school buildings, building trust with school district administration school principals and other school rules and regulations that need to be followed for the implementation and sustainability of the project.

3. Sensor Fabrication, Calibration and Deployment: The students were provided with components and instructions to assemble the sensor package within the classroom. At its core was a Raspberry Pi 3, a Wi-Fi-enabled single-board computer (hereafter, just “Pi”). Air quality sensors included three gaseous sensors from Alphasense Ltd. (Great Notley, United Kingdom): model CO-A4F for carbon monoxide (CO), model OX-A431 for the combined measurement of ozone and nitrogen dioxide (O_3_ and NO_2_, respectively), and model NO2-A43F for NO_2_. For PM measurements, we implemented the Shinyei PPD42NJ sensor (Shinyei Technology Co., Ltd.; Kobe, Japan) due to its widespread market penetration at the time. We also included an Aosong Electronics Co., Ltd. (Guangzhou, China) DHT22 digital relative humidity and temperature sensor. The initial goal was to have these sensor packages operate autonomously from the roofs of school buildings. This provided two design challenges for the Davidson students: 1) integrate a solar panel/battery array with the Pi to provide power; and 2) prototype and fabricate protective housing to protect the sensors from the weather. Eventually, the solar panel was not used due to technical challenges (e.g., Pi not turning on after recharging the battery) and electrical outlets in the wall were used instead.
4. Data Analytics: Acquisition and processing was initially conducted using code developed by OSU faculty and students. Custom code was written in the Python3 programming language to record and store sensor data locally on Pi and to transmit the data to Google’s cloud-based storage platform, Firebase. Code written in the R programming language was used to retrieve data from the cloud for subsequent use in data processing and web-based visualization using the ShinyR package. Also, students wrote, tested and documented their code for real-time data collection and visualization using Google Sheets, an online Microsoft Excel-like program. The OSU researchers oversaw maintaining and updating the cloud-computing solution and the web-based application for data analytics and visualization.

5. Translation: Several outreach activities were conduction both in schools and in the community based on early results from this project. Examples of outreach activities within schools included student-led presentations about the project to other teachers during a school district-wide professional development day and “Shark Tank”-style presentations within Davidson High School. Outreach activities in the community included setting up an information table on the project at the local county fair and Earth Day event, giving interviews about the project to a local newspaper and the City of Hilliard Environmental Sustainability Commission. Some of these activities were conducted in partnership between students and researchers.

Although this project is ongoing, the order of processes described above remained the same for each year of the project with slight variations and different levels of effort and focus by students. These variations and their impact on the project timeline and lessons learned are described in the next section.

## Results and discussion

**Fig. 4:**
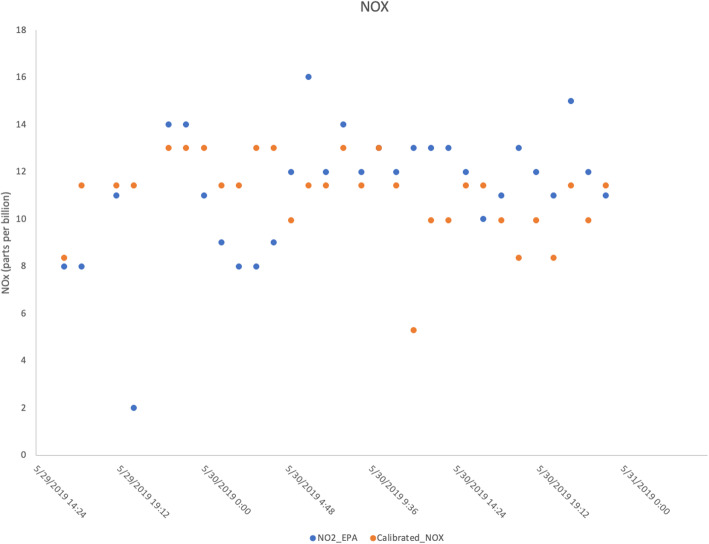
Comparison of NOx measured by EPA land-based monitors and calibrated NOx values measured by low-cost air quality sensor deployed at Davidson High School

**Fig. 5:**
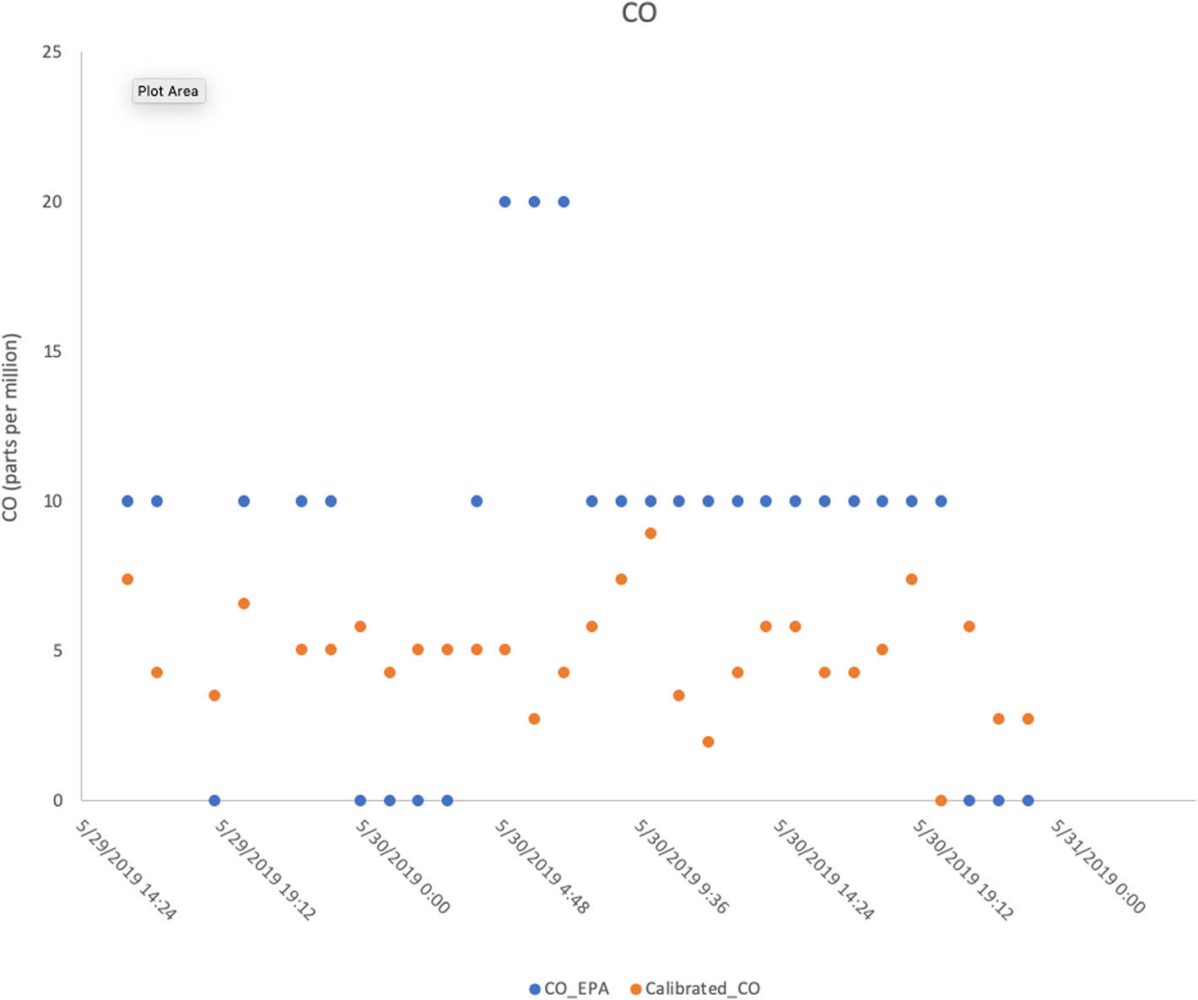
Comparison of CO measured by EPA land-based monitors and calibrated CO values measured by low-cost air quality sensor deployed at Davidson High School

Overall, the project engaged with 14 high school students over 4 years and continues to remain engaged with the EDD course at Davidson High School (Table 1). The primary products generated through the project included a website that displayed data acquired from deployed sensors (beta version available at http://airhilliard.bmi.osumc.edu, Fig. 3 for screenshot) and a GitHub-like repository for the Python code used to log data from the sensors and transmit data to the Google Cloud Firebase platform (available at https://bitbucket.org/airquality/airqualityosu/src/master/). Preliminary results showed a good comparison between the sensor-based and EPA land-based monitor data for both CO (Fig. 4) and NOx (Fig. 5).

**Table 1:** Description of high school students involved and their areas of focus in each year

School year	# of students engaged	Focus areas for students involved	Products generated by the partnership
2016–2017	4	Locations for sensor deployment; Permission from school district administration and principals; Community and School District Outreach	Beta version of a web-based application for data visualization; presentation on the project to teachers at the district-wide professional day event
2017–2018	4	Sensor package design and fabrication; Solar panel and Wi-Fi integration and testing; Sensor calibration	
2018–2019	3	Sensor enclosure design, fabrication and testing; Sensor calibration; Sensor deployment; Data collection in Google Sheets; Community Outreach	Article in a science communication publication (not peer-reviewed); articles in local newspapers and web media^1,2,3,4^
2019–2020	3	Mass production of sensor enclosure; Sensor deployment; Community and School District Outreach	Work currently in progress

1“High School Students Join Ohio State Professors in Citizen Science Project” Published 2018. URL: https://ceg.osu.edu/news/2018/12/high-school-students-join-ohio-state-professors-citizen-science-project

2“Dublin’s Emerald Campus: Sensors may offer answers, education”. Published in 2019. URL: https://www.thisweeknews.com/news/20190129/dublins-emerald-campus-sensors-may-offer-answers-education

3“Davidson students assisting in air-quality program” Published 2018. URL: https://www.thisweeknews.com/news/20181023/eye-on-environment-davidson-students-assisting-in-air-quality-program

4“Using Low Cost Sensors and Citizen-Science to examine Air Quality” Published 2019. URL: http://cdn.researchoutreach.org/Flipbooks/RO108/index.html#

While the focus areas of each cohort were slightly different in each year of the project (see Table 1) the end goal remained the same: deployment of a low-cost air quality sensor network across multiple school buildings in Hilliard, OH. Each year a different cohort of students built upon the work of the previous cohort of students. In some cases, cohorts scrapped and did not use the previous cohort’s plans (e.g., design of an enclosure for sensors, wiring) and started from the beginning (e.g., 2018–2019 cohort completely re-designed the sensor enclosure originally designed by the 2016–2017 cohort). The EDD teacher remained the same for the entire project period.

There were several reasons for the longer-than-expected duration and variation in focus area by each cohort of students. First, a shared objective of academic researchers and the EDD teacher at Davidson at the beginning of the project was that students would learn multiple aspects of engineering design and translational research through this project. In practice, this meant minimal involvement of academic researchers with students except where domain expertise, for example in exposure assessment, public health and sensor package fabrication, were necessary for the success and sustainability of the project. While this slowed progress, it empowered the students with the freedom to work independently, a substantial benefit in their academic development.

Second, the self-selection process for each year’s cohort led to a lack of continuity in ideas and diversity in group dynamics each year. These two issues resulted in each cohort of students approaching the design, fabrication and other challenges in slightly different ways. However, this reinforces the concept of iteration in the engineering design process that the students learn in their EDD curriculum. This suggests that each subsequent cohort thought critically about prior cohorts’ efforts, maintaining elements that worked well and making improvements where needed.

Third, there was frustration on part of the high school teacher in two areas: i) creating curricular activities based on data collected from the air quality sensors and ii) longer than the expected duration of the project. The source of frustration was identified by the academic faculty as their inability to comprehend the curricular activity design process. For example, the high school teacher’s desired learning outcomes for the activity required that data from the sensors be validated against EPA’s land-based monitors and the website should provide background information on each air toxicant. These challenges were eventually overcome by redesigning the website to the desired specifications of the teacher’s vision and engaging with the teacher outside of the project. One way the academic faculty engaged outside the scope of the project was by serving as judges in the Davidson Engineering Shark Tank competition where students from the EDD course presented group projects. This type of “leaning in” by academic faculty provided useful insights for academic faculty that they were able to apply to the academic-community partnership continuously. The academic faculty have been participating in the Shark Tank competition for the past 3 years and intend to continue their participation in future years.

Lastly, there were technical issues. Changes to local area network infrastructure within Davidson, which was modified between Years 2 and 3, resulted in the loss of Wi-Fi connectivity at the same location on the roof the previous year. Challenges with solar panels arose, possibly due to some combination of larger-than-expected power draw by the Pi and sensors, limited solar radiation in Ohio during the academic year, and lack of expertise in connecting the solar panels/battery to the Pi. As a result, the placement of sensor packages is limited to locations with accessible wired connections for network and power. This suggests that unexpected issues, which often arise in citizen-science based research projects, has the potential to be leveraged for other types of learning that were not planned for in the original research project. The lesson learned is to allow for extra time in the overall timeline during future projects due to the high-risk, high-reward nature of the work.

Despite these challenges, several unequivocal successes were resulting from this project including direct contact between academic researchers with cohorts of pre-engineering high school students on annual basis, the extension of the academic-high school partnership model to two additional school districts near Columbus, OH and the experience students gained in research under the supervision of OSU faculty. Also, some of the project alumni are now students at OSU and have been engaged as undergraduate researchers early in their academic careers with an academic faculty member’s research lab.

The nature of the interdisciplinary collaboration in the academic setting (i.e., between A.H. in Public Health and A.A.M. in Environmental Engineering) was critically linked to the above-mentioned challenges and successes of the academic-community partnership (Fig. 1). We identified three factors that contributed to this linkage. First, we leveraged the strengths of each academic discipline to achieve the aims of the project. The focus of public health research, in general, lends itself naturally to engaging with community members since public health aims to improve the health and well-being of populations. On the other hand, engineering research, in general, lends itself naturally to engaging with individual clients since its focus is on designing and implementing solutions to engineering problems. This citizen science-based project was successful because the perspectives and expertise from both disciplines were equally valued and utilized within the project. Other researchers should pay close attention to disciplinary boundaries to maximize the impact of interdisciplinary collaborations. One example of how we stayed within our disciplinary boundaries was by providing input for students only in the areas and topics that we each knew well based on training and experience. Second, open communication was critical on an ongoing basis. Open communication through regular meetings (bi-weekly) between the academic researchers and asking questions and clarifying concepts from each discipline also facilitated interdisciplinary research within the academic setting. Also, setting and revising expectations together about workload and time commitments between faculty members helped to create an environment conducive to sustaining the interdisciplinary collaboration. Third, regularly incorporating feedback from community partners (i.e., Davidson students and teachers) into the research activities helped to also ensure success. Regularly incorporating feedback from Davidson students and teachers allowed the academic researchers to re-calibrate their level of engagement based on the needs of each cohort of students. In years that school outreach and community engagement were the focus area, A.H. was more actively engaged with students whereas in years that sensor design and fabrication were the focus areas, A.A.M. was more actively engaged with students. The duration of this project was longer than expected due to this back and forth between focus areas of each cohort, the time it took for each researcher to “lean-in” to each other’s discipline, and the trial-and-error nature of citizen science projects. Academic researchers and the Davidson teacher decided early in the project that the students would be driving the implementation of the project with advice and expertise from academic researchers on an as-needed basis. Although this decision led to a longer period to achieve the aims of the citizen-science based project, it was a key reason for continuing the project because incremental progress was evident after each cohort of students who were engaged through this project.

The aspect of this project with the most room for improvement and a future research direction currently being explored within our research team is related to the technology. Low-cost sensor technologies, especially for PM, are undergoing a rapid growth that offers several opportunities for conducting Translational Data Analytics in Environmental Health; improved sensor technologies can facilitate improvements to the sensor packages. For example, many older models of PM sensors (including the Shinyei PPD) relied on thermal buoyancy to drive sample flow through the sensor; however, many newer sensors include a small internal fan. These advancements make it easier for citizen-science projects that focus on environmental health issues to use low-cost sensors with high confidence in their accuracy. Moreover, additional sensors could be integrated into the existing sensor package, which only contains sensors for traffic-related air pollutants. For example, in urban or near-road environments, there may be an interest in monitoring ambient noise, which has been linked to negative health outcomes [21]. Similarly, an ambient light sensor could be added to quantify incoming solar radiation, which may be of interest in and of itself but could also aid in future efforts towards the use of solar panels [22]. The implementation of additional sensors makes this project a good use case for smart cities or intelligent communities, who are increasingly looking for problems to solve and this project because this project focuses on technology-enabled solutions. Other citizen science projects that share similar features and aims as the project described in this study include the Array of Things project [23] and IVAN (Identifying Violations Affecting Neighborhoods) Air Monitoring [7].

Based on our description of the project, we have provided a logic model that captures the essential inputs, activities, outputs and outcomes in a more general manner (Table 2). This logic model serves as a framework that others may leverage for establishing similar academic-community partnerships for conducting Translational Data Analytics in Environmental Health.

**Table 2:** Logic model for the framework of Translational Data Analytics in Environmental Health

Needs/ Rationale	Inputs	Activities	Outputs	Short-term Outcomes	Long-term Outcomes
Lack of air quality data in microenvironments (Hilliard, OH)	Low-cost air quality sensors High School teacher(s) and students (Seniors) enrolled in Engineering Design and Development Course. Expertise in environmental health, environmental engineering, sensor calibration and data analytics. Graduate students to perform sensor calibration and assist with computer programming.	**Introduction (Phase I):** -Academic faculty introduced to each other and set expectations for collaboration -Academic faculty introduced to a high school teacher(s) and discuss academic-community partnership and set partnership goals, expectations and timelines -Academic faculty introduced to high school students and meet to introduce the project **Education (Phase II):** -Academic faculty educate each other about disciplinary perspectives and set boundaries based on training and experience -Academic faculty learn from high school teacher about curricular objectives and share their expertise with high school students **Sensor Fabrication, Calibration and Deployment (Phase III):** -Students assemble sensors, fabricate sensor housing and identify potential locations for siting; -Academic faculty provides expertise to students and facilitates sensor calibration **Data Analytics (Phase IV):** -Academic faculty provided computer programs/code for submitting data from sensors to internet cloud-based storage and maintained web-based application for visualizing and analyzing data from sensors -High School teacher and students provided feedback on the design and analytics features of the web-based application **Translation (ongoing across phases):** -All members of the partnership participated in local media interviews about the project -presented project goals and progress -Students introduced the project to teachers in the school district through presentations at professional development settings -Students raised awareness about air pollution and health impacts at local Earth Day events	1. Clear communication plan, timeline and information sharing platform (Google Drive) between all partners. 2. Sustained partnerships involving multiple cohorts of students throughout the project. 3. Multiple fabrication plans for sensor housing; schematics and working versions of the integrated circuit board for Raspberry Pi, sensors and cables. 4. Design matrix for selecting schools based on multiple objectives for exposure assessment, data collection, and socioeconomic factors. 5.Open-source code for data logging from sensor to cloud-based storage. 6. A working version of a ShinyR web-based application to visualize air quality data.	Raise awareness about air pollution levels in Hilliard, OH. Offer hands-on training opportunities for students at the intersection of public health and engineering. Build sustainable academic-community partnerships. Deploy a working version of a low-cost air quality sensor.	Expand low-cost air quality sensor network to 10 or more school building in the Hilliard School District Disseminate curricular materials based on an existing partnership that consists of plans to implement each phase of the partnership in a scalable manner. Add additional sensors to the module, such as noise, and maintain current sensor networks through the extension of the academic-community partnership. Continuous validation of low-cost sensors with federal reference monitors.

In conclusion, we have outlined a framework for Translational Data Analytics in Environmental Health and discussed how to implement the framework by describing a citizen-science based air quality sensor project. Lessons learned from this project include: 1) the need for sustained funding because project timelines are a function of community needs/capacity and building interdisciplinary rapport in the academic setting and 2) the need for a dedicated staff person to manage the academic-community relationships. Future community engagement and research activities in this project will focus on refining the implementation of the framework, evaluating its effectiveness in terms of impacts on environmental exposures and health outcomes, and defining and measuring metrics for the value of translation in the context of data analytics and environmental health.

## Data Availability

The datasets during and/or analyzed during the current study available from the corresponding author on reasonable request.
